# Enterococcal PrgU Provides Additional Regulation of Pheromone-Inducible Conjugative Plasmids

**DOI:** 10.1128/mSphere.00264-21

**Published:** 2021-06-09

**Authors:** Lena Lassinantti, Martha I. Camacho, Rebecca J. B. Erickson, Julia L. E. Willett, Nicholas R. De Lay, Josy ter Beek, Gary M. Dunny, Peter J. Christie, Ronnie P.-A. Berntsson

**Affiliations:** a Department of Medical Biochemistry and Biophysics, Umeå University, Umeå, Sweden; b Department of Microbiology and Molecular Genetics, McGovern Medical School, Houston, Texas, USA; c Department of Microbiology and Immunology, University of Minnesota, Minneapolis, Minnesota, USA; d Wallenberg Centre for Molecular Medicine, Umeå University, Umeå, Sweden; University of Iowa

**Keywords:** type 4 secretion systems, conjugation, regulation

## Abstract

Efficient horizontal gene transfer of the conjugative plasmid pCF10 from Enterococcus faecalis depends on the expression of its type 4 secretion system (T4SS) genes, controlled by the P_Q_ promoter. Transcription from the P_Q_ promoter is tightly regulated, partially to limit cell toxicity caused by overproduction of PrgB, a T4SS adhesin. PrgU plays an important role in regulating this toxicity by decreasing PrgB levels. PrgU has an RNA-binding fold, prompting us to test whether PrgU exerts its regulatory control through binding of *prgQ* transcripts. We used a combination of *in vivo* methods to quantify PrgU effects on *prgQ* transcripts at both single-cell and population levels. PrgU function requires a specific RNA sequence within an intergenic region (IGR) about 400 bp downstream of P_Q_. PrgU interaction with the IGR reduces levels of downstream transcripts. Single-cell expression analysis showed that cells expressing *prgU* decreased transcript levels more rapidly than isogenic *prgU*-minus cells. PrgU bound RNA *in vitro* without sequence specificity, suggesting that PrgU requires a specific RNA structure or one or more host factors for selective binding *in vivo*. PrgU binding to its IGR target might recruit RNase(s) for targeted degradation of downstream transcripts or reduce elongation of nascent transcripts beyond the IGR.

**IMPORTANCE** Bacteria utilize type 4 secretion systems (T4SS) to efficiently transfer DNA between donor and recipient cells, thereby spreading genes encoding antibiotic resistance as well as various virulence factors. Regulation of expression of the T4SS proteins and surface adhesins in Gram-positive bacteria is crucial, as some of these are highly toxic to the cell. The significance of our research lies in identifying the novel mechanism by which PrgU performs its delicate fine-tuning of the expression levels. As *prgU* orthologs are present in various conjugative plasmids and transposons, our results are likely relevant to understanding of diverse clinically important transfer systems.

## INTRODUCTION

**FIG 1:**
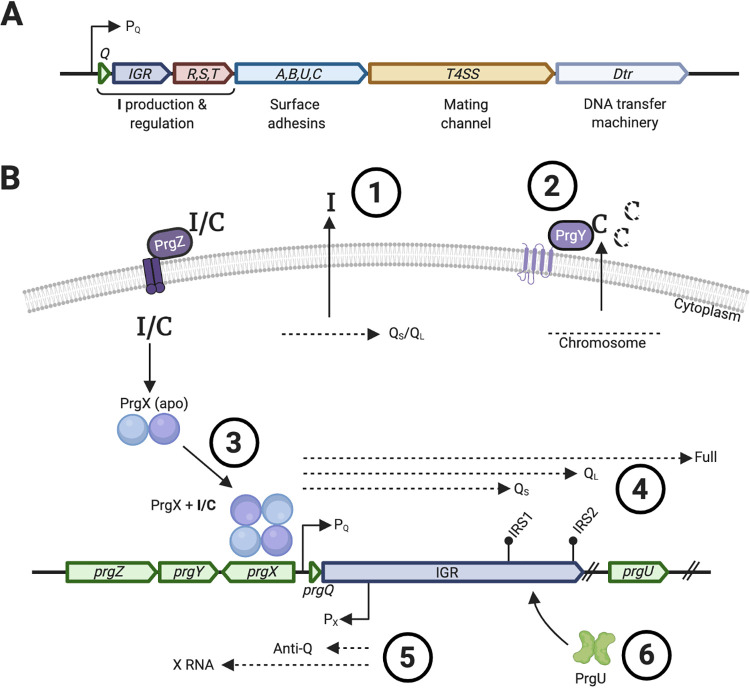
Schematic overview of the *prgQ* operon and its regulation. (A) Schematic illustration of the genetic organization of the entire *prgQ* operon (not to scale). Note that *prgU* is situated within the cassette containing the surface adhesin, even though PrgU belongs to the regulatory proteins. (B) The inhibitory peptide I is transcribed from the pCF10 plasmid (#1), whereas the inducing C peptide is transcribed from the genome of all E. faecalis cells (#2). Both I and C peptides are secreted to the outside of the cell, where the C peptide from donor cells gets partially degraded by the PrgY protease (#2) (38, 39). Both I and C peptides are subsequently bound by PrgZ and imported via a permease (23). The transcriptional regulator PrgX is a dimer in its apo state but tetramerizes upon binding either I or C peptide. Depending on which pheromone is bound, it either represses transcription of the *prgQ* operon (PrgX/I) or induces it (PrgX/C) (#3) (22, 38, 40). Induction of the *prgQ* operon produces 3 transcripts, Q_S_, Q_L_ and Full (#4) (40). The *prgX* operon also produces two transcripts, one of which is the anti-Q RNA that aids the formation of a terminator structure of the *prgQ* operon at the inverted repeat sequence 1 (IRS1) (#5) (7). In uninduced cells, anti-Q levels are sufficient to interact with all nascent *prgQ* transcripts, favoring formation of the IRS1 structure and transcript termination. Pheromone induction leads to the production of many more *prgQ* transcripts, which then overwhelm the pool of anti-Q. In the unpaired *prgQ* transcripts, IRS1 does not form, and transcription extends through the rest of the operon. The findings that we present in this article show that PrgU interacts with a region in the IGR, in between IRS1 and IRS2, thereby decreasing the expression of proteins transcribed from downstream genes (#6).

Hospital-acquired (nosocomial) infections and antibiotic resistance are among the largest threats to global health according to the World Health Organization (1). Pathogens often acquire their resistance genes via transferable plasmids and other mobile genetic elements (2). A common opportunistic pathogen in nosocomial infections is the Gram-positive bacterium Enterococcus faecalis. Many clinical isolates of E. faecalis harbor conjugative plasmids. These plasmids contain type 4 secretion systems (T4SS) that are responsible for their own horizontal gene transfer. They most often also contain genes encoding resistance to antibiotics and various virulence factors. One of the best-studied E. faecalis plasmids is pCF10, a member of the superfamily of pheromone-responsive plasmids. pCF10 codes for resistance to tetracycline from an acquired Tn*925* transposon, and its *prgQ* operon of ∼30 kb encodes cell surface adhesins including PrgB (also termed aggregation substance [AS]) as well as the entire T4SS (Fig. 1A) (3, 4).

The regulation of this system has been the topic of several reviews (3, 4), and an overview is shown in Fig. 1B. Several negative regulatory checkpoints are involved. Transcription of the *prgQ* operon from the P_Q_ promoter is governed by the ratio of two hydrophobic peptide sex-pheromones: cCF10 (called peptide C, for clumping; sequence: LVTLVFV) and iCF10 (called peptide I, for inhibiting; sequence: AITLIFI). Peptides I and C counteractively modulate the DNA binding activity of PrgX and, in turn, initiation of transcription from P_Q_. The intergenic region (IGR) positioned between *prgQ* and the *prgRST* genes carries two potential transcription terminators, IRS1 and IRS2. These two terminator sites, especially IRS2, have previously been indicated to be relatively weak (5). Early studies also identified potential secondary structures resembling 23S and 16S rRNA helices in the IGR, which could serve as binding targets for ribosomal proteins (6). Northern blot analysis of pheromone-induced donor cells shows 2 predominant RNA species, Q_S_ and Q_L_, whose 3′ termini correspond to the position of IRS1 and IRS2, respectively, as well as extended transcripts that include the entire operon (7). A countertranscript RNA termed anti-Q regulates formation of the IRS1 terminator structure (7), and it is likely that IRS1 and IRS2 could also stabilize transcripts subject to degradation via 3′-to-5′ RNase activity, contributing to the total pool of these RNAs. Recently, a small (13-kDa) protein encoded by *prgU* has been shown to play a critical role in controlling overexpression of the *prgQ* operon (8).

PrgU has a pseudouridine synthase and archaeosine transglycosylase (PUA)-like fold as shown by its crystal structure (PDB: 2GMQ). PUA domains are conserved RNA binding motifs and can be found in all three kingdoms of life (9). They are often found in proteins involved in posttranscriptional modifications of tRNA and rRNA and in proteins involved in ribosome biogenesis and translation (10). Mutations in PUA motifs are reported to be involved in human diseases such as cancer and dyskeratosis congenita (11, 12). The length of the PUA domains usually varies between 64 and 96 amino acids. In most known PUA-containing proteins, these RNA binding motifs are joined to a catalytic domain responsible for RNA modifications (13–15). In contrast, the PUA fold encompasses the entire PrgU sequence (119 amino acids), although PrgU has short insertions in a few loop regions not found in other PUA domains. Deletion of *prgU* from pCF10 leads to increased levels of the proteins encoded by the *prgQ* operon (Fig. 1B). This includes PrgB, which is toxic to the cells at high levels (8). Prolonged pheromone induction of pCF10Δ*prgU* mutants is therefore lethal, and the rare survivors contain suppressor mutations that display a noninducible phenotype (pCF10::*prgU*^Res^) (8). It has been suggested that other factors, such as the PrgR/PrgS proteins or bacterial host proteins, could coordinate with PrgU to regulate the expression of the *prgQ* operon (8).

Using a combination of *in vivo* and *in vitro* assays, we show that PrgU regulates expression of *prgQ* operon-carried genes. It does so by interacting with a specific sequence within the IGR, just downstream of the IRS1 sequence. Our cumulative data indicate that this interaction depends on another unidentified plasmid- or host-encoded factor(s). In bacteria, regulatory mechanisms controlling abundance of transcripts are well documented at the levels of transcription initiation and elongation/termination. Regulation at the levels of message stability and mRNA processing has also been well documented, although the details of these mechanisms are not as thoroughly understood. This report describes new information about the mechanism by which PrgU prevents toxic overexpression of a conjugation protein by degrading or inhibiting synthesis of transcripts extending past the IGR during induction of donor cells by a peptide sex pheromone.

## RESULTS

### PrgU suppresses expression of the *prgQ* operon.

**FIG 2:**
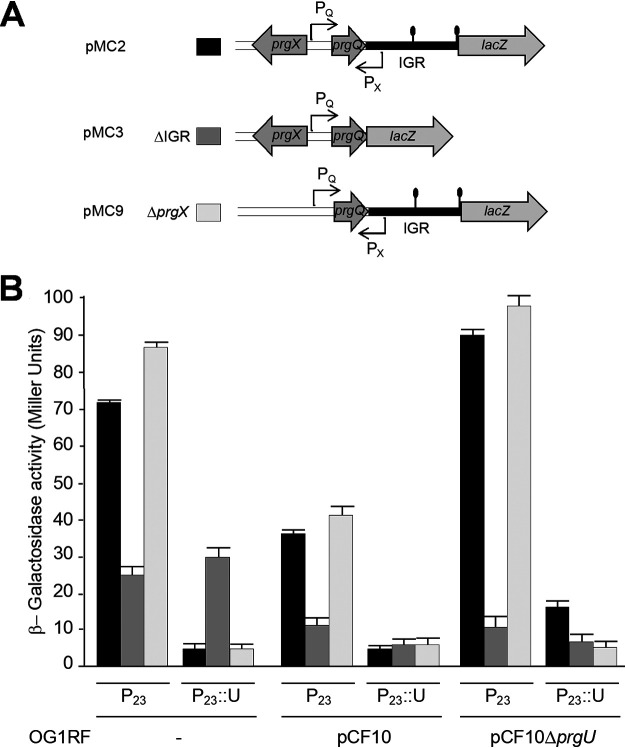
PrgU inhibition of gene expression depends on the intergenic region (IGR) and is independent of PrgX. (A) Schematic overview of the plasmids used in this study. The pMC plasmids carry the P_Q_ promoter with its regulatory region and the transcriptional *lacZ* reporter. This reporter gene is positioned at the start site of *prgR* in pMC2 and pMC9 (Fig. 1B) and at the 3′ end of *prgQ* in pMC3. In pMC9 the *prgX* transcriptional repressor upstream from the P_Q_ promoter is deleted. (B) β-Galactosidase activities originating from expression of the *lacZ* reporter gene on the pMC plasmids listed in panel A (coded in grayscale) in OG1RF cells without a pCF10 plasmid (−) or with wild-type pCF10 or pCF10Δ*prgU*. These cells also contain the pDL278p23 vector (P_23_) or this vector constitutively expressing *prgU* (P_23_::U). *n* = 3 independent biological replicates, and the error is the standard deviation.

Because PrgU adopts a PUA fold implicated in binding rRNA or tRNA, we hypothesized that PrgU might exert its regulatory control by binding RNA structural folds within the IGR. To test this model, we first constructed reporter plasmids carrying the P_Q_ promoter and a downstream *lacZ* reporter gene. One plasmid (pMC2) contains the IGR in between the promoter and *lacZ*, while another (pMC3) lacks the IGR. A third plasmid (pMC9) contains the IGR (like pMC2) but lacks the *prgX* gene (Fig. 2A). We then monitored *lacZ* expression from these plasmids in induced OG1RF cells (−) or OG1RF cells harboring pCF10 or pCF10Δ*prgU* by a β-galactosidase activity assay (Fig. 2B). These strains also carried the vector pDL278p23, which has a constitutive P_23_ promoter (designated P_23_) or a variant of this plasmid, pMB11, that constitutively expresses *prgU* (designated P_23_::U).

OG1RF cells with pMC2, which contains the IGR (Fig. 2A), and the P_23_ vector plasmid exhibited robust β-galactosidase activity. Isogenic strains that also contained P_23_::U had only background levels of reporter activity (Fig. 2B, black bars). Introduction of pCF10 into OG1RF(pMC2, P_23_) resulted in lower β-galactosidase activity, presumably due to PrgU production from native *prgU*. Reporter activity remained at background levels in cells carrying pCF10 and P_23_::U due to abundant PrgU production. (Fig. 2B, middle, black bars). As expected, the mitigating effect of pCF10 on β-galactosidase activity was not observed upon introduction of pCF10Δ*prgU* into OG1RF(pMC2, P_23_); in fact, this strain exhibited elevated β-galactosidase activity relative to OG1RF lacking pCF10 altogether (Fig. 2B, left and right, black bars).

In cells with pMC3, which lacks the IGR (Fig. 2A), PrgU production from either pCF10 or P_23_::U had no effect on β-galactosidase activities (Fig. 2B, compare dark gray bars, P_23_ and P_23_::U). Cells harboring pMC3 and pCF10 did exhibit slightly reduced β-galactosidase activities compared to cells with only pMC3. However, this effect was independent of PrgU, as pCF10Δ*prgU* caused a similar reduction of *lacZ* expression from pMC3 (Fig. 2B). We also observed that cells with pMC9, which lacks *prgX*, exhibited slightly higher β-galactosidase activity levels in the absence of PrgU overproduction, compared to pMC2, which carries *prgX* (compare P_23_ black and light gray bars). This was expected because, in contrast to pMC2-carrying cells, the pMC9-carrying cells completely lack the transcriptional regulator PrgX. This lack of PrgX gives rise to a high constitutive transcription level of the P_Q_ operon partly due to less anti-Q (16), in the OG1RF background (−), and lower PrgX levels in cells carrying coresident pCF10 or pCF10Δ*prgU*. Importantly, β-galactosidase activity from the pMC9 plasmid was still strongly reduced to near-background levels by PrgU overproduction (Fig. 2B, compare light gray bars, P_23_ and P_23_::U). Taken together, these results establish that PrgU is dependent on the presence of the IGR to inhibit expression of the downstream gene(s) and that PrgU acts independently of PrgX.

### Effects of PrgU on Q transcripts *in vivo*.

**FIG 3:**
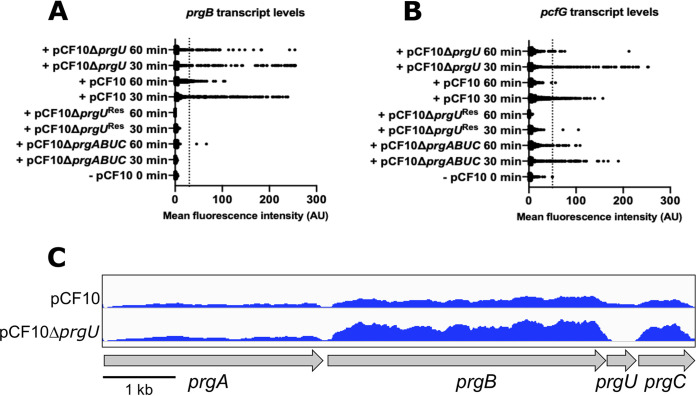
Effects of PrgU on pCF10 transcription at the population and single-cell levels. (A and B) HCR analysis of *prgB* and *prgG* transcripts in single cells as a quantification of the mean fluorescence intensity per cell of 500 random cells, using hybridization probes against *prgB* (A) and *pcfG* (B) transcripts. Thresholds (thin dotted lines) used are 30 AU and 50 AU, respectively. Transcription was induced with (+) 10 ng/ml peptide C (cCF10 pheromone) for 30 or 60 min. Uninduced cells (−) at 0 min and pCF10Δ*prgU*^Res^-containing cells (which cannot be induced) were used as negative controls. pCF10Δ*prgABUC*-containing cells are a negative control for *prgB*. (C) mRNA reads in the *prgA* to -*C* region in induced pCF10 wild-type and pCF10Δ*prgU* cells. The height of the blue lines indicates the transcription level in each strain. The complete RNA-seq data for this experiment are available in Data Sets S1 and S2.

To evaluate the effect of PrgU on *prgQ* transcript levels, we used single-cell *in situ* hybridization chain reaction (Fig. 3A and B). This technique both facilitates quantitative comparison of the levels of different transcripts in the same cell and determines the range of variation of expression dynamics within a population that is exposed to the same stimulus (17). The mRNA levels of fluorescently labeled *prgB*, an early gene in the operon behind the P_Q_ promoter, and *pcfG*, a late gene in the same operon (11.5 kbp downstream of *prgB*), were examined in single cells. OG1RF cells carrying the following plasmids were analyzed: pCF10, pCF10Δ*prgU*, pCF10Δ*prgU*^Res^, and pCF10Δ*prgABUC*. pCF10Δ*prgU*^Res^ has an additional mutation (*prgR*Δ271) that prevents C peptide-dependent induction of P_Q_ transcription (8), and it served as a negative control together with uninduced cells (−, pCF10 in Fig. 3A and B). pCF10Δ*prgABUC* was used as an additional background control for *prgB*, as this variant lacks the *prgB* gene, as well as *prgA*, *prgU*, and *prgC*.

Cells with a mean fluorescence intensity higher than the threshold for either *prgB* or *pcfG* (Fig. 3A and B; thin dotted line, 30 and 50 absorbance units [AU], respectively) were considered to be induced. As expected, in the negative-control strains there was no fluorescence observed for the *prgB* probe (with the exception of two of the 500 randomly chosen pCF10Δ*prgABUC* cells at 60 min), and very little for the *pcfG* probe (only two of the 500 randomly chosen pCF10Δ*prgU*^Res^ cells above the threshold at 30 min). Also as expected, many pCF10Δ*prgABUC* cells displayed high fluorescence intensities for *pcfG*. These controls established that there is no overlap between the two hybridization probes and/or fluorescence channels, consistent with previous findings (18, 19).

To study the effect of PrgU on *prgB* and *pcfG* transcript levels, we compared the levels of fluorescence in cells with pCF10Δ*prgU* to those in cells with wild-type pCF10. The cells from the pCF10Δ*prgU* strain had a tendency to aggregate and showed an increase in cell lysis upon induction, likely due to increased levels of PrgB as previously reported (8, 20). This complicated the measurements in this strain and probably led to an underestimation of the number of induced cells (Table 1). However, the pCF10Δ*prgU* cells that were induced, and not aggregated, contained a larger amount of *pcfG* transcripts and kept those high levels for a longer time than wild type (compare pCF10 and pCF10Δ*prgU* at 60 min, Fig. 3B). The same trend, but not as prominent, can also be observed for *prgB* transcripts (Fig. 3A).

**TABLE 1:**
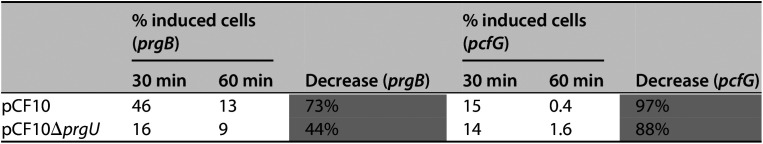
Percentage of cells that have transcript levels of *prgB* or *pcfG* above the threshold, at 30 and 60 min after induction (from Fig. 3A and B)^*a*^

a The shaded columns show the decrease of cells above threshold at 60 min compared to 30 min, as a percentage. Note that the number of induced cells is probably underestimated for the pCF10Δ*prgU* strain (see main text).

We observed that all pCF10-carrying cells with high levels of *pcfG* transcripts also had high *prgB* transcript levels, except for the pCF10Δ*prgABUC*-containing cells that lack *prgB*, whereas not all cells with high *prgB* levels had high *pcfG* levels (example shown in Fig. S1 in the supplemental material). This was noted previously and could be due to the fact that it takes the RNA polymerase roughly 7 min to traverse from the *prgB* gene to the *pcfG* gene (18).

10.1128/mSphere.00264-21.1 FIG S1 Cells induced with 10-ng/ml C peptide (cCF10) that express early transcripts also express late transcripts after 60 min. Transcripts (early gene [*prgB*] or late gene [*pcfG*]) are labeled by HCR with either Alexa Fluor 647 (red, left panel and merge image) or Alexa Fluor 488 (green, middle panel and merge image); nucleic acids were labeled with Hoechst 33342 (blue, shown only in merge image on the right) to highlight individual cells (blue). Right column contains a red-green merge with the Hoechst stains for all cells (blue). The strain pCF10Δ*prgU*^Res^ is used as a negative control since it cannot be induced by the C peptide. Bars, 10 μm. Download FIG S1, TIF file, 2 MB. Copyright © 2021 Lassinantti et al. 2021 Lassinantti et al. https://creativecommons.org/licenses/by/4.0/ This content is distributed under the terms of the Creative Commons Attribution 4.0 International license.

Additionally, PrgU-mediated reduction of *prgB* transcripts in induced cells was also supported by transcriptome sequencing (RNA-seq) analysis of total mRNA from donor cells subjected to a 60-min induction by cCF10 pheromone (Fig. 3C and Data Sets S1 and S2). At this time point, wild-type cells have already progressed into the shutdown phase of the pheromone response as shown in the hybridization chain reaction (HCR) analysis (Fig. 3A and B) and by previous findings (8, 21). However, this RNA-seq data suggested that cells lacking PrgU retained about twice as much *prgB* mRNA as was observed for wild-type cells. We note that analysis of *prgA* transcript levels in these experiments is complicated by the fact that this gene and the adjacent *prgT* gene are subject to a significant basal level of transcription from a constitutive promoter located at the 5′ end of *prgT*.

**FIG 4:**
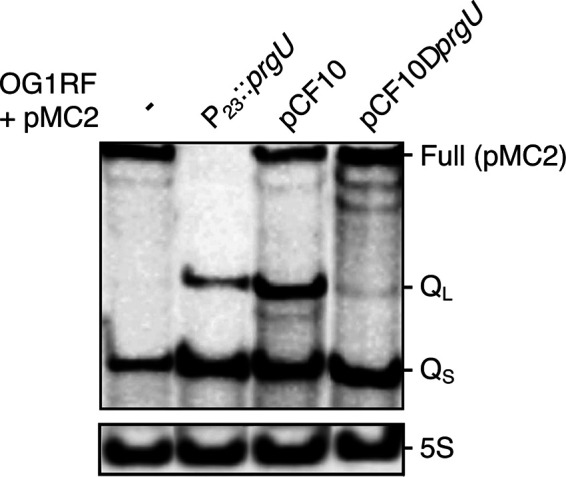
PrgU expression reduces the formation of full-length [Full (pMC2)] transcript and leads to formation of Q_L_ and, at high concentrations, an increase in Q_S_ transcripts. Northern blot analyses of total RNA extracts probed with an oligonucleotide specific for the 5′ end of the IGR or 5S RNA as a loading control. All strains analyzed were OG1RF cells containing the pMC2 plasmid, induced by addition of exogenous C peptide. In one strain, no additional plasmid was present (−). The other strains contained either the pMB11 vector that constitutively expresses *prgU* (P_23_::*prgU*), wild-type pCF10, or pCF10Δ*prgU*. Note that Q_S_ and Q_L_ are also formed from the pCF10 plasmids, while the full-length transcript produced from pCF10 is much larger and not visible here.

We further investigated the effect of PrgU on *prgQ* operon transcript levels with Northern blot assays. As shown in Fig. 1, transcription from P_Q_ results in the formation of three transcripts, Q_S_ and Q_L,_ whose 3′ termini correspond to the IRS1 and IRS2 structures in the IGR, respectively, and the full-length transcript encoding the surface adhesins and T4SS. We sought to compare Q_S_, Q_L_, and full-length transcript levels, but because the full-length transcript from pCF10 could not be resolved due to its length, we instead analyzed transcripts generated in strains harboring pMC2. This enabled detection of the full-length *lacZ* transcript as a proxy for the transcript of the full *prgQ* operon (Fig. 2A). In total RNA extracts of OG1RF(pMC2) cells lacking *prgU*, the Q_S_ and full-length [Full (pMC2)] transcripts were detected, but not Q_L_ (Fig. 4, first lane). However, in extracts from cells expressing PrgU, either from the P_23_ promoter or from the pCF10 plasmid, the Q_L_ transcript was detected. Quantification of the bands showed that the amount of the Q_S_ transcript in P_23_::*prgU*, pCF10, and pCF10Δ*prgU* increased 3-fold compared to OG1RF + pMC2. Full-length pMC2 transcripts were no longer detected in the strain that overproduced PrgU from the P_23_ promoter, and the level of this transcript was reduced by ∼40% in the strain with wild-type pCF10.

**FIG 5:**
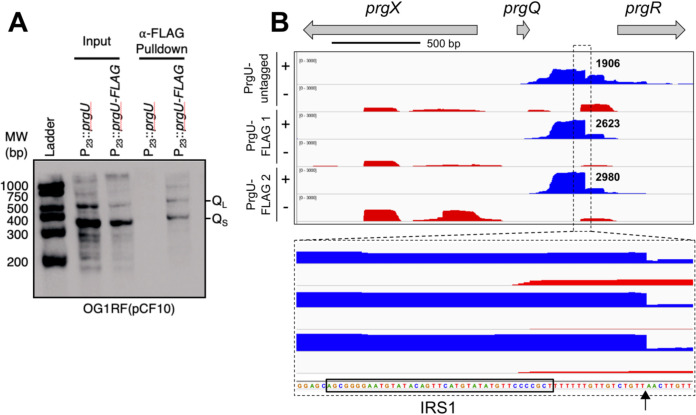
PrgU-FLAG binds to the Q_L_ and part of the Q_S_ transcripts. Cell lysates from C peptide-induced OG1RF(pCF10) strains carrying either pMB11 (expresses PrgU from the P_23_ promoter, P_23_::*prgU*) or pMC10 (expresses FLAG-tagged PrgU from the P_23_ promoter, P_23_::*prgU-FLAG*) were mixed with magnetic beads coated with FLAG antibodies. (A) Samples from the total cell lysates (input) and the elution fractions from the washed beads (pulldown) were subjected to Northern blot analysis using an oligonucleotide protein probe specific for the 5′ end of IGR RNA. Transcripts corresponding to the size of Q_S_ and Q_L_ bound to the magnetic beads in the presence of FLAG-tagged PrgU but not with the nontagged control. Note that full-length transcripts of the *prgQ* operon from pCF10 are much larger and not visible here. MW, molecular weight. (B) RNA-seq analysis of the RNA from PrgU pulldown fractions from lysates of either C peptide-induced (+) or noninduced (−) cells. PrgU-FLAG1 and -2 represent two biologically separate pulldowns. The top panel graphically depicts the relative read counts in the *prgQ* region, whereas the lower panel shows an enlargement with the RNA sequence. The sequence corresponding to IRS1 is highlighted by a black box, and the putative 3′ end of the prominent pulldown product is indicated by an arrow.

Taken together, these data suggest that PrgU blocks the formation or enhances the breakdown of RNA transcripts downstream of IRS1/2. Since PrgU has a PUA fold that is predicted to bind RNA with rRNA-like structures, we hypothesized that PrgU binds Q_L_, which contains the whole IGR with the IRS1 and IRS2 site and possibly also to Q_S_, which contains over half of the IGR including the IRS1 site. To test this hypothesis *in vivo*, we conducted a pulldown experiment with total cell extracts of OG1RF(pCF10) strains engineered to produce FLAG-tagged PrgU or nontagged PrgU as a control. FLAG-tagged PrgU was shown to be functional as it complements OG1RF(pCF10::Δ*prgU*). RNA recovered from the affinity pulldowns was subjected to Northern blot analysis with a probe designed to detect Q_S_ and Q_L_. As seen in Fig. 5A, both Q_S_ and Q_L_ transcripts were identified in the pulled-down material in the presence of FLAG-tagged PrgU but, importantly, not with the nontagged PrgU control. By densitometry, we determined that the amounts of Q_L_ transcripts were the same in the input and affinity-enriched sample, but the level of Q_S_ was reduced 5-fold in the affinity-enriched sample compared to the input. This suggested that FLAG-PrgU bound and pulled down more Q_L_ (or extended Q transcripts processed back to Q_L_) than Q_S_.

In separate pulldown experiments, we precipitated the affinity-enriched RNA for analysis by RNA-seq (Fig. 5B and Data Set S2). The data showed significant RNAs from the IRS1 to IRS2 regions in all samples. In particular, a very prominent RNA species with a deduced 3′ end was detected, located 15 bp downstream from the 3′ end of the IRS1 sequence (note that the IRS1 structure is not generated in nonterminated *prgQ* transcripts). An RNA containing the same 3′ end was also seen in the nontagged sample, at slightly lower levels, but not significantly enriched in the pulldown samples. At present, the biological significance of this RNA is not clear, but it could represent a stable processing product of a PrgU-dependent degradation pathway; this RNA would not likely be detected in the Northern analysis because its 5′ region does not extend to the sequence complementary to the Northern blot probe. Whether generation of this RNA requires PrgU will be an important question for future mechanistic studies. We note that RNA concentrations from PrgU-FLAG samples were ∼10-fold greater than that of the untagged negative controls. This suggests enrichment of PrgU-bound RNAs relative to background material by FLAG-independent attachment to the beads used for pulldowns.

10.1128/mSphere.00264-21.5 DATA SET S1 RNA-seq data showing the differential expression of pCF10 transcripts in OG1RF pCF10 and OG1RF pCF10Δ*prgU*. Download Data Set S1, XLSX file, 0.03 MB. Copyright © 2021 Lassinantti et al. 2021 Lassinantti et al. https://creativecommons.org/licenses/by/4.0/ This content is distributed under the terms of the Creative Commons Attribution 4.0 International license.

10.1128/mSphere.00264-21.6 DATA SET S2 RNA-seq data showing transcripts identified from PrgU-FLAG pulldown experiments. Download Data Set S2, XLSX file, 0.02 MB. Copyright © 2021 Lassinantti et al. 2021 Lassinantti et al. https://creativecommons.org/licenses/by/4.0/ This content is distributed under the terms of the Creative Commons Attribution 4.0 International license.

### *In vitro* characterization of PrgU.

**FIG 6:**
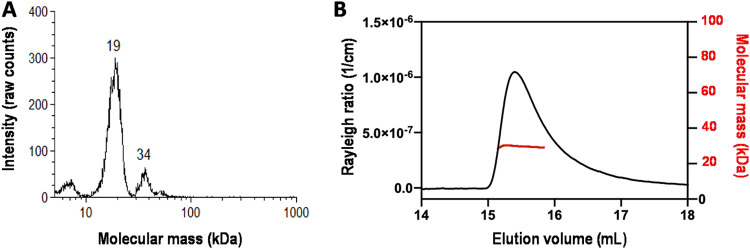
Oligomeric state of PrgU. (A) GEMMA of 0.01 mg/ml PrgU. The determined molecular masses are shown above the peaks. (B) SEC-MALS profile of PrgU, loaded at a protein concentration of 1 mg/ml, showing a molecular mass of 29 kDa over the range of the peak.

The results of our studies described above suggested that PrgU interacts with the Q transcripts. To test this directly, we overproduced and purified PrgU to characterize its biochemical properties in isolation. PrgU, with an N- or C-terminal His tag (15.9 kDa), was produced in Escherichia coli and purified to homogeneity using a 2-step purification protocol. Both His-PrgU and PrgU-His eluted at the same volume on the size exclusion chromatograph (SEC). We then assessed the oligomeric state of purified PrgU-His by GEMMA (gas-phase electrophoretic molecular mobility analysis) and SEC-MALS (size exclusion chromatography-multiangle light scattering). The GEMMA indicates that PrgU-His exists as both a dimer and a monomer (Fig. 6A). The SEC-MALS experiment was done with a much higher protein concentration (∼1 mg/ml versus 0.01 mg/ml for the GEMMA) and shows almost exclusively a dimer form of PrgU-His (Fig. 6B). Taken together, these data indicate that PrgU is in a concentration-dependent monomer-dimer equilibrium.

**FIG 7:**
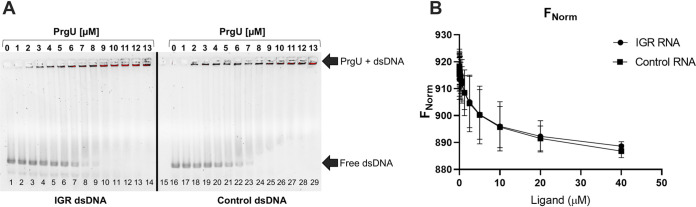
PrgU binding to IGR. (A) EMSA with dsDNA of the IGR or an equally long control dsDNA with increasing concentrations of PrgU-His (0 to 13 μM). In all cases, 100 nM dsDNA was used along with IGR (lanes 1 to 14) or control (lanes 16 to 29). Lanes 1 and 16 contain only 100 nM DNA, ds-IGR or ds-control, respectively, and lane 15 contains only the highest concentration of protein used in this assay (13 μM PrgU). (B) Data from MST experiment with a constant concentration of His-labeled PrgU (50 nM) and a varied concentration (between 0 and 40 μM) of the nonlabeled binding partner (RNA IGR and control RNA). *n* = 3 independent measurements; error bars represent the standard deviation.

We assayed whether purified PrgU binds double-stranded DNA (dsDNA) by electrophoretic mobility shift assays (EMSAs). The results showed that PrgU binds a dsDNA substrate corresponding to the IGR with low-micromolar affinity but that it has a similar affinity for the control dsDNA substrate (Fig. 7A and Fig. S2). Next, we investigated the binding of PrgU to single-stranded DNA (ssDNA), dsDNA, and RNA forms of the IGR, as well as to shorter sequences corresponding only to the IRS1 and IRS2, by microscale thermophoresis (MST) (Table S1). However, under the tested experimental conditions, we were unable to detect binding to IRS1 or IRS2 (for neither RNA, ssDNA, nor dsDNA). We did observe binding to the longer IGR RNA, but PrgU bound equally well to control RNA of similar length (Fig. 7B). Our data therefore suggest that PrgU has low-micromolar affinity to both dsDNA and RNA but that binding is not sequence specific under our experimental conditions.

10.1128/mSphere.00264-21.2 FIG S2 EMSA with dsDNA of the IGR or an equally long control dsDNA with increasing concentrations of His-PrgU (0 to 13 μM). In all cases, 100 nM dsDNA was used along with IGR (lanes 1 to 14) or control (lanes 16 to 29). Lanes 1 and 16 contain only 100 nM DNA, ds-IGR, or ds-control, respectively, and lane 15 contains only the highest concentration of protein used in this assay (13 μM PrgU). Download FIG S2, TIF file, 1.1 MB. Copyright © 2021 Lassinantti et al. 2021 Lassinantti et al. https://creativecommons.org/licenses/by/4.0/ This content is distributed under the terms of the Creative Commons Attribution 4.0 International license.

10.1128/mSphere.00264-21.3 TABLE S1 Oligonucleotides used for PrgU interaction assays. Download Table S1, DOCX file, 0.01 MB. Copyright © 2021 Lassinantti et al. 2021 Lassinantti et al. https://creativecommons.org/licenses/by/4.0/ This content is distributed under the terms of the Creative Commons Attribution 4.0 International license.

## DISCUSSION

Regulation of the conjugative plasmid pCF10 by pheromone sensing has been extensively studied (3, 4, 22, 23). The results have revealed a multitude of mechanisms to regulate the expression from the P_Q_ promoter (3, 4, 21, 22, 24, 25). More recently, it was discovered that PrgU, a previously uncharacterized protein, is also involved in this regulation. Bhatty et al. (8, 20) showed that induction with the C peptide was toxic to cells carrying a pCF10 plasmid lacking *prgU* (pCF10Δ*prgU*). This toxicity was relieved by extragenic suppressor mutations or by PrgU overproduction, both of which blocked the production of Prg adhesins. Levels of PrgA, PrgB, and PrgC were increased in cells with pCF10Δ*prgU*, and conversely, levels were strongly reduced upon *prgU* overexpression. These findings led to the proposal that PrgU integrates with one or more of the P_Q_ repression systems to control the synthesis of the Prg surface adhesins upon pheromone induction (8). Importantly, *prgU* genes are widely spread on both plasmids and chromosomes in enterococci and are most often found together with a *prgB* gene (8). The information about PrgU presented here is thus not solely linked to the pCF10 plasmid, but the PrgU function likely plays an important role in various other plasmids and strains.

Here, we showed that PrgU reduces expression from genes downstream of the IGR (Fig. 2). In our reporter fusion studies and Northern blot analyses, we utilized *lacZ* reporter plasmids that lacked all *prg/pcf* genes downstream of the IGR. The observed effects of PrgU thus required only the P_Q_ promoter, *prgQ*, and the IGR and no other gene products from the *prgQ* operon, including PrgR and PrgS, which were previously postulated to coordinate with PrgU to control P_Q_ transcription (Fig. 2 and 4) (8). Since none of the reporter plasmids contained a *prgU* gene (Fig. 2), these experiments also confirmed that expression in *trans* allows for full PrgU function. We also showed that PrgU regulation was unaltered by the presence or absence of *prgX* (Fig. 2), indicating that PrgU does not exert its negative control through effects on formation, stability, and/or function of the PrgX/C or PrgX/I complexes. From our experiments, we cannot rule out whether PrgU enhances P_X_ transcription or leads to increased levels of the anti-Q transcript, but in view of our data we deem these options unlikely. Based on our findings, we therefore propose that PrgU functions independently of other known regulators of the pCF10 pheromone response system.

PrgU’s control of expression of the *prgQ* operon depends on the presence of the IGR positioned between the P_Q_ promoter and the regulated genes (Fig. 2). PrgU was observed to have a PUA-like fold (8), which is often involved in RNA binding and found in proteins that play a role in posttranscriptional modifications of tRNA and rRNA. This was of special interest, given previous evidence for control of *prgB* expression at both transcriptional and translational levels (6). The data from these previous experiments suggested that ribosomes, sequences in the IGR upstream from IRS1, and a 5′ untranslated region between *prgA* and *prgB* are all involved in a novel translational regulatory mechanism unique to *prgB*. Conceivably, this poorly understood mechanism for regulation of PrgB synthesis could be related to the specific effect of PrgU on reduction of *prgB* mRNA and PrgB protein levels.

In addition to data from the transcriptional reporter studies, several other experimental findings support our working model that PrgU exerts its negative control through interaction with RNAs produced from the IGR. We showed that PrgU reduces full-length transcript levels by three independent methods by measuring (i) the transcript levels from an early gene and a late gene from the *prgQ* operon by HCR analysis (Fig. 3A and B) and (ii) the mRNA levels of *prgB* by RNA-seq data (Fig. 3C) and by (iii) Northern blot analysis of the RNA from a reporter plasmid (Fig. 4). The Northern blot analysis also showed that PrgU overproduction leads to an accumulation of Q_S_ and Q_L_ transcripts, presumably at the expense of the full-length transcript (Fig. 4). Results of the affinity pulldown assays further showed that both of these transcripts (especially Q_L_) copurified with FLAG-tagged PrgU (Fig. 5A). Furthermore, RNA-seq analysis of the affinity-enriched RNA suggests that a predicted linear region just downstream of the IRS1 sequence may be the actual binding target for PrgU (Fig. 5B). We speculate that this RNA was generated by PrgU binding to this region just downstream of IRS1 on longer transcripts (note that the IRS1 terminator structure is unlikely to have been formed in these transcripts). Bound PrgU would then block 3′-to-5′ degradation by host RNases at this binding site. Alternatively, PrgU binding to this site on nascent transcripts could also inhibit further elongation via effects on IRS2 RNA polymerase processivity, which would lead to a similar outcome. We also observe the terminated transcript in the control pulldown with nontagged PrgU (Fig. 5B). It is important to note that untagged PrgU is also overexpressed in the nontagged cells and thus produces the same terminated transcript as in the cells with FLAG-tagged PrgU. However, as mentioned earlier, the pulldown with the FLAG-PrgU contained ∼10 times more RNA than the control. Thus, we believe that much of the signal from the untagged samples presented in Fig. 5 likely resulted from nonspecific binding to the resin.

Both the reporter constructs (pMC2 and pMC9 shown in Fig. 2) that were negatively regulated by PrgU contain the predicted PrgU binding site. Interestingly, the essential target region of PrgU is upstream from the *prgU* gene. Such an arrangement could facilitate a timing delay, where PrgU-mediated negative regulation can take place only after a period of protein expression. This would first enable the production of adequate levels of the conjugation machinery for efficient plasmid transfer, followed by a reduction in transcript levels to prevent protein levels rising further and becoming toxic for the donor cells. This is supported by our HCR analysis (Table 1 and Fig. 3A and B).

Despite several lines of evidence suggesting that PrgU interacts with a specific RNA target sequence in the IGR, we were unable to confirm that purified PrgU specifically bound the IGR mRNA *in vitro*. EMSAs with dsDNA and MST data with RNA of the IGR and control sequences indicated that PrgU nonspecifically binds to both dsDNA and RNA with an affinity in the low-micromolar range (Fig. 7A and B). The affinity and specificity were the same with both N- and C-terminally tagged PrgU. We also know that FLAG-tagged PrgU can complement a Δ*prgU* mutant *in vivo*. This indicates that the purification tag did not affect binding (compare Fig. 7A and Fig. S2 in the supplemental material). The observed low-affinity and sequence-nonspecific binding to dsDNA could indicate that PrgU is concentrated around DNA, perhaps associated with active RNA transcription elongation complexes. This would allow it to quickly bind to the specific IGR binding site once it is produced to block further RNA synthesis or to recruit nucleases involved in RNA turnover. We suspect that specificity for RNA of the IGR could be imparted by another PrgU binding partner, which is missing in our *in vitro* experiments. The candidates include one or more host-encoded regulators or even RNA polymerase itself. Another possibility, raised by Bhatty et al. (8), is that PrgU stabilizes the interaction of anti-Q RNA or another regulatory RNA with Q transcripts (41), which we did not test for in our *in vitro* experiments. Alternatively, it is possible that PrgU stabilizes the IRS1 and IRS2 terminator structures and in that way inhibits the production of the full-length transcript. We also determined that PrgU exists in a concentration-dependent monomer/dimer equilibrium in solution, which might be related to its biological function. PrgU dimerization might enable binding of two RNA target sequences as a prerequisite for effective regulatory control of downstream transcription or might be necessary for recruitment of coregulatory protein(s) or antisense RNA.

In summary, we have supplied several lines of evidence supporting our model that PrgU interacts with IGR mRNA and in so doing strongly reduces the accumulation of full-length transcripts. Although various mechanisms can be posited to account for PrgU’s inhibition of full-length transcription, three of the more likely ones are (i) transcriptional blockade, (ii) strengthening the IRS1 and IRS2 terminator structures, and (iii) recruitment of RNase(s) for selective degradation. Experiments to determine the exact mechanism of PrgU, as well as the presumptive protein or RNA cofactor, remain exciting avenues for future research.

## MATERIALS AND METHODS

### E. faecalis strains, growth, and pheromone induction.

The bacterial strains, plasmids, and oligonucleotides used in the study are listed in Tables S1 and S2 in the supplemental material. For plasmid construction, E. coli DH5α served as a host. E. coli strains were grown in Lysogeny broth (LB) (Difco Laboratories) at 37°C with shaking. E. coli strains were grown with the following antibiotics as needed: chloramphenicol (20 μg ml^−1^), erythromycin (100 μg ml^−1^), and spectinomycin (50 μg ml^−1^). E. faecalis strains were grown in brain heart infusion broth (BHI; Difco Laboratories) or M9 minimal medium (26) at 37°C without shaking. E. faecalis strains were grown with the following antibiotics as needed: erythromycin (100 μg ml^−1^ final concentration for plasmid markers, 10 μg ml^−1^ for chromosomal markers), fusidic acid (25 μg ml^−1^), rifampin (200 μg ml^−1^), spectinomycin (1,000 μg ml^−1^ for plasmid markers, 250 μg ml^−1^ for chromosomal markers), streptomycin (1,000 μg ml^−1^), tetracycline (10 μg ml^−1^), and chloramphenicol (10 μg ml^−1^). Antibiotics were obtained from Sigma-Aldrich.

10.1128/mSphere.00264-21.4 TABLE S2 Strains, plasmids, and oligonucleotides used in this study. Download Table S2, DOCX file, 0.02 MB. Copyright © 2021 Lassinantti et al. 2021 Lassinantti et al. https://creativecommons.org/licenses/by/4.0/ This content is distributed under the terms of the Creative Commons Attribution 4.0 International license.

### Plasmid constructions.

pMC1 is the shuttle vector plasmid pCI372 carrying *prgX* to *prgA* from pCF10 followed by *lacZ* with its own Shine-Dalgarno sequence. It was constructed by PCR amplification of the region of the IGR beginning at a natural XbaI site through the end of *prgA* using pCF10 as a template and amplification of *lacZ* from pCJK205. The two PCR products were digested with XbaI-BamHI and BamHI-SphI, respectively, and the resulting products were introduced into XbaI/SphI-digested plasmid pCIE.

pMC2 is pCI372 carrying *prgX* through the end of the IGR followed by *lacZ*. It was constructed by inverse PCR using pMC1 as a template and the primers listed in Table S2. pMC3 is pCI372 carrying *prgX* through the end of *prgQ* followed by *lacZ*. It was constructed by inverse PCR using pMC1 as a template and the primers listed in Table S2. pMC9 is pMC2 deleted of *prgX*. It was constructed by inverse PCR using pMC2 as a template and the primers listed in Table S2. pMC10 is plasmid pDL278p23 expressing *prgU-FLAG* from the P_23_ promoter; it was constructed by inverse PCR using pMB11 as a template (8) and the primers listed in Table S2. pMC11 is plasmid pDL278p23 carrying the intergenic region (IGR) of pCF10; it was constructed by amplification of the IGR from pMC2, digestion with BamHI and SphI, and insertion into similarly digested pDL278p23.

To produce the His-tagged PrgU overexpression vectors for protein purification, p10-mini (8) was used as a template for PCR amplification. The *prgU*-containing PCR products were cloned into the initial cloning vector pINIT using SapI. The constructs were then transferred into p7XC3H or p7XNH3 via the FX cloning system (27).

### β-Galactosidase assays.

β-Galactosidase activity was assayed as previously described (28) with the following modifications: cells were cultured overnight in M9-yeast extract (YE) with selective antibiotics. The overnight culture was diluted 1:5 in fresh medium with 50 ng/ml cCF10 and grown for 90 min to mid-log phase. The cells were pelleted and resuspended in assay buffer (Z buffer: 60 mM Na_2_HPO_4_·7H_2_O, 40 mM NaH_2_PO_4_·H_2_O, 10 mM KCl, 1 mM MgSO_4_, 50 mM β-mercaptoethanol, pH set to 7.0) to eliminate error due to the effects of different carbon sources in the growth medium on the β-galactosidase enzyme activity. Error bars indicate the standard deviation of three independent measurements.

### Single-cell *in situ* hybridization chain reaction (HCR).

E. faecalis OG1RF cells were used with 4 different plasmids: pCF10, pCF10*ΔprgU*, pCF10Δ*prgU*^Res^, and pCF10Δ*prgABUC* (Table S2). Overnight cultures were diluted 1:10 in M9 minimal medium (optical density at 600 nm [OD_600_] of ∼0.2) and grown to log phase (OD_600_ of 1.2). The cultures were induced with 0 or 10 ng/ml cCF10 and harvested after 0, 30, or 60 min.

Paraformaldehyde (PFA) with a final concentration of 4% was used to fix the cells followed by incubation overnight at 4°C before resuspending in phosphate-buffered saline (PBS) buffer supplemented with 2.7 mM KCl containing RNaseOUT (Invitrogen). HCR labeling was done as described before (19). In short, the cells were permeabilized and HCR labeling was done on cell suspensions. The hybridization probes were designed against an early gene (*prgB*) and a late gene (*pcfG*). The probes and hairpin amplifiers were obtained from Molecular Instruments (www.molecularinstruments.com). The B2H1 and B2H2 and the B5H1 and B5H2 amplifiers conjugated to Alexa Fluor 647 and 488 were used to detect *prgB* and *pcfG* transcripts. Cells were counterstained with Hoechst 33342.

Cells were then mounted as previously described (19). Images were acquired using a Zeiss Axio Observer.Z1 confocal microscope with an LSM 800-based Airyscan superresolution detector system (Zeiss). Confocal images were acquired through a 63× 1.40-numerical-aperture (NA) objective (Zeiss) as z stacks at approximately 0.79-μm intervals using Zen software (version 2.1; Zeiss). Images were deconvolved and flattened using a maximum intensity projection (MIP) with Ortho Display, followed by image analysis by MATLAB (R2019b; MathWorks). For HCR analysis, the blue Hoechst fluorescence reference channel was used to determine pixel locations of individual cells and colocalized fluorescent overlap from HCR-labeled transcripts was quantified. The processed images (deconvolved, flattened by MIP, and subjected to background subtraction) were imported into MATLAB (R2019b; MathWorks) in a 16-bit TIFF format and analyzed as described previously (19). The reference channel images were binarized using Otsu’s method (28) for thresholding. Objects between 3 and 50 pixels in size were analyzed as cells in the following analysis. HCR intensity value corresponding to each cell was calculated by taking the mean intensity of the pixels corresponding to each cell. To normalize the samples, 500 random cells from each image were used. The induction thresholds were decided based on the expression levels in the uninduced pCF10 population for *pcfG*, while for the threshold determination of *prgB*, the pCF10Δ*ABUC* mutant was used. Graphs were created using GraphPad Prism 8.0.

### Total RNA extraction from E. faecalis.

E. faecalis strains were grown overnight in 5 ml M9 medium without antibiotics at 37°C. The culture was then diluted to an OD_600_ of ∼0.03 in 50 ml M9 medium and grown without shaking to an OD_600_ of 0.1. Pheromone (cCF10) was added at a final concentration of 20 ng/ml, and cultures were grown to an OD_600_ of 0.3. Cells were pelleted by centrifugation at 16,000 × *g* at 4°C for 15 min. The pellet was resuspended in 1 ml of RNA-pro solution from a Fast RNA Blue kit (MP Biomedicals), and the mixture was transferred to a Fast Prep tube containing lysing matrix B. Cells were disrupted by bead beating five times at a setting of 400 rpm for 40 s with 1- to 2-min intervals on ice. Insoluble material was removed by centrifugation at 16,000 × *g* for 5 min at 4°C, and the supernatant (∼750 μl) was transferred to a fresh microcentrifuge tube and stood for 5 min at room temperature. After chloroform extraction, precipitation with absolute ethanol, and a final wash with cold 75% ethanol (made with diethyl pyrocarbonate [DEPC]-treated H_2_O), the RNA pellet was air dried and resuspended in 100 μl of DEPC-H_2_O. The RNA concentration was determined using a NanoDrop spectrophotometer, and RNA was stored at −80°C.

### Northern blot analysis.

RNA samples (2 μg) were loaded onto 10% Criterion Tris-buffered EDTA (TBE)–urea precast gels (Bio-Rad) and electrophoresed at 70 V until the dye front reached the bottom of the gel. RNA samples were transferred to a Zeta-Probe GT membrane (Bio-Rad) using a Trans-Blot SD semidry transfer apparatus (Bio-Rad) according to the manufacturer’s guidelines. Transferred RNA was UV cross-linked and hybridized overnight with 100 ng/ml of 5′-biotinylated probes (Table S2) in ULTRAhyb hybridization buffer (Ambion) at 42°C as described previously (29, 30). Blots were developed using the BrightStar BioDetect kit protocol (Ambion) and imaged with a ChemiDoc MP imager (Bio-Rad). Quantifications of the bands were performed by densitometry tracing using Image J software.

### PrgU-FLAG affinity pulldowns.

E. faecalis strains were grown overnight in BHI (5 ml) and then diluted to an OD_600_ of ∼0.03 in 200 ml BHI and grown without shaking to an OD_600_ of 0.1. cCF10 was added at a final concentration of 20 ng/ml, and cultures were grown to an OD_600_ of 0.3. Cells were pelleted by centrifugation at 16,000 × *g* at 4°C for 5 min, washed once with 10 ml Tris-buffered saline (TBS) buffer (50 mM Tris-HCl, pH 7.4; 150 mM NaCl), resuspended in 1 ml TBS, and transferred to a 1.5-ml Eppendorf tube. Cells were pelleted by centrifugation at 16,000 × *g* at 4°C for 5 min, the supernatant was removed, and the cell pellet was flash frozen in a dry ice-ethanol slurry and stored at −80°C.

Cell lysates were prepared by suspending cells in 500 μl of TBS buffer with 5 μl of Halt protease inhibitor cocktail and 2 μl of SUPERase. Cells were added to a prechilled tube containing 500 μl of glass beads (0.1 mm) and disrupted by repeated cycles of maceration for 40 s at 400 rpm with intervals on ice. TBS buffer (500 μl) was added, and the slurry was vortexed for 30 s, after which cell fragments and other material were pelleted by centrifugation at 12,000 × *g* for 30 min at 4°C. The supernatant was transferred to a prechilled tube, the volume was increased to 1 ml with cold TBS buffer, and 5 μl of SUPERase inhibitor was added.

An aliquot representing the input fraction was removed, and the remaining material was subjected to FLAG pulldowns by addition of 75 μl of anti-FLAG magnetic bead slurry to a 2.0-ml Dolphin tube (Costar 3213). The mixture was then placed on a nutator (rocker) for 2.0 h at 4°C, after which time the resin was pelleted by centrifugation at 4,000 × *g* for 30 s at 4°C. The supernatant was removed, and the resin was washed 3 times with 1.5 ml of TBS buffer, with incubations on a nutator for 1 min, and then subjected to magnetic bead pulldowns at 4°C. Material bound to the FLAG beads was eluted by addition of 15 μl of 5-μg/μl 3×FLAG peptide solution and 250 μl of 3×FLAG elution buffer. Samples were incubated on a nutator at 4°C for 30 min and then centrifuged for 30 s at 4,000 × *g*. The supernatant containing the eluted material was transferred to a new tube.

TBS (250 μl) and 3 M sodium acetate, pH 5.2 (50 ml), were added to the input and the eluted fractions (made as described above), and the RNA was isolated by acid phenol-chloroform extraction. The aqueous phase was transferred to a new tube containing 500 μl of neutral phenol-chloroform–isoamyl alcohol, followed by centrifugation at 14,000 rpm for 15 min. The supernatant was then mixed with an equal volume of isopropanol and placed at −80°C overnight. RNA was pelleted by centrifugation at 14,000 rpm for 15 min, washed once with 75% ethanol (EtOH), and air dried at room temperature. RNA was suspended in 20 μl DEPC-treated water and stored at −80°C until analysis via Northern blotting (see above).

### RNA-seq analyses.

For whole-cell RNA-seq, overnight bacterial cultures of OG1RF(pCF10) and OG1RF(pCF10Δ*prgU*) were diluted 1:20 in BHI medium and grown for 1 h at 37°C. Cells were then induced with 10 ng/ml cCF10 followed by incubation for 1 h at 37°C (31, 32). rRNA was depleted using a MICROBExpress kit following manufacturer’s instructions.

For RNA-seq on pulldowns, overnight cultures (OG1RF pCF10 carrying either pCIE::*prgU* or pCIE::*prgU*-FLAG) were subcultured to an OD_600_ of 0.03 in BHI. At an OD_600_ of 0.1, cCF10 (20 ng/ml) was added. Cultures were grown to an OD_600_ of 0.3 and pelleted (16,000 × *g* at 4°C for 10 min). Cells were washed once with 10 ml 1× TBS and once with 1 ml 1× TBS, frozen on dry ice, and stored at −80°C. Cell pellets were resuspended in 500 μl TBS with 5 μl HALT protease inhibitor cocktail (ThermoFisher) and 2 μl SUPERase and then added to a prechilled tube containing 500 μl glass beads (0.1 mm). Tubes were placed in a BeadBug microtube homogenizer (Benchmark Scientific) for 5 cycles (40 s, 400 rpm), incubating on ice between cycles. Five hundred microliters TBS was added to each tube, and tubes were pelleted at 12,000 × *g* for 30 min at 4°C. The supernatant was transferred to a prechilled microcentrifuge tube containing 5 μl SUPERase. Fifty microliters was removed as a protein sample, and 50 μl was removed for an “input” RNA sample. The remaining volume was incubated with 75 μl prewashed anti-FLAG slurry in a 2.0-ml tube for 2 h at 4°C. The resin was pelleted (4,000 × *g* for 30 s at 4°C) and washed with 1.5 ml 1× TBS three times. FLAG-tagged bacterial proteins were eluted with 3×FLAG peptide as follows. Two hundred fifty microliters elution buffer (15 μl of 5-μg/μl 3×FLAG peptide in 500 μl TBS) was added to the resin and incubated with rotation at 4°C for 30 min. After centrifuging the resin (4,000 × *g* for 30 s at 4°C), the supernatant (containing eluted protein) was transferred to a new tube to which 50 μl 3 M sodium acetate (pH 5.2) was added. RNA was extracted with 1 volume acid phenol-chloroform. The aqueous phase was transferred to a new tube containing 500 μl neutral phenol-chloroform–isoamyl alcohol (25:24:1), extracted with 1 volume isopropanol, precipitated at −80°C overnight, and washed with 1 volume 75% ethanol. The pellet was dried and resuspended in 20 μl DEPC-treated water.

All RNA samples were treated with Turbo DNase (ThermoFisher; rigorous method described by the manufacturer) and submitted to the University of Minnesota Genomics Center for Illumina library prep. Whole-cell RNA samples were prepared using a nonstranded TruSeq RNAv2 kit, and libraries were size selected to generate inserts of 200 bp. Pulldown samples were processed using the Illumina TruSeq stranded mRNA preparation kit. To retain small RNAs, size selection was not performed between library preparation and sequencing for pulldown samples. All samples were sequenced as paired-end reads on an Illumina HiSeq 2500 in high output mode (125-bp read length for whole-cell samples and 50-bp read length for pulldown samples). Sequencing files were trimmed to remove contaminating adapter sequences and low-quality bases using Trimmomatic. Reads were mapped to the pCF10 reference sequence (NC_006827) using Rockhopper (33, 34) and visualized using IGV (35). Rockhopper generates normalized expression values similar to reads per kilobase per million (RPKM) for all transcripts. The RNA-seq data, including RPKM and expression values, can be found (as Rockhopper output files) in Data Sets S1 and S2 in the supplemental material.

### Protein production and purification.

Both variants of PrgU, with either N- or C-terminal 10×His tag and a 3C protease site, were produced in E. coli BL21(DE3) and grown in terrific broth (TB) medium in a LEX bioreactor (Epiphyte). The cells were grown at 37°C to an OD_600_ of 1.0 to 1.5, and the temperature was then lowered to 18°C before induction with 0.5 mM IPTG (isopropyl-β-d-thiogalactopyranoside). The cells were incubated for 18 h at 18°C before being harvested via centrifugation and lysed in lysis buffer (50 mM KPi [pH 7.8], 150 mM NaCl, 15 mM imidazole [pH 7.8], and 10% glycerol) using a Constant cell disruptor (Constant Systems) with a pressure of 25 kilopounds/in^2^. Cell lysates were run over an immobilized metal-ion affinity chromatograph (IMAC) with Ni-nitrilotriacetic acid (NTA) beads using gravity flow and washed with 20 column volumes (CV) of wash buffer (50 mM KPi [pH 7.8], 500 mM NaCl, 50 mM imidazole [pH 7.8]) followed by 5 CV of wash buffer containing 2 M LiCl followed again by 20 CV of wash buffer. The proteins were then eluted with elution buffer (50 mM KPi [pH 7.0], 500 mM NaCl, and 500 mM imidazole [pH 7.0]). Eluted material was run over a size exclusion chromatograph (SEC) in 20 mM KPi (pH 7.0), 150 mM NaCl using either an S200 10/300 GL Increase (GE Healthcare) or an S75 10/300 GL (GE Healthcare) column.

### GEMMA.

Gas-phase electrophoretic mobility macromolecule analysis (GEMMA) on PrgU-His was performed as previously described (36). Briefly, the main peak of PrgU-His after SEC was collected and diluted to a concentration of 0.01 mg/ml in 20 mM ammonium acetate, pH 7.8. The sample was scanned 3 times to increase the signal-to-noise ratio.

### Size-exclusion chromatography coupled to multiangle light scattering (SEC-MALS).

Two hundred microliters of purified PrgU-His at 1 mg/ml was run over a Superdex 200 10/300 GL Increase column on an ÄKTA Pure system in line with Wyatt Treos II (light scattering) and Wyatt Optilab T-Rex (refractive index) in 20 mM potassium phosphate (pH 7.0), 150 mM NaCl. Astra software (Wyatt Technology; version 7.2.2) was used to collect and analyze the SEC-MALS data.

### DNA and RNA oligonucleotides used for *in vitro* binding studies.

The template used for producing the DNA of the IGR and the control was pCF10 or pRS01, respectively (Table S2). A touchdown PCR was done with Phusion polymerase and a starting annealing temperature of 72°C with a decrease of 1°C/cycle. The PCR products were cloned into pRAV23 (Addgene) using EcoRI and HindIII restriction sites and transformed into Top10 cells. The cells were grown followed by miniprep (Qiagen) for plasmid isolation. The plasmids were digested with EcoRI (Thermo Fisher) and HindIII (Thermo Fisher) using the Fast Digest buffer (Thermo Fisher) and used as a template for T7 *in vitro* transcription. The T7 reaction mixture consisted of 9 mM MgCl_2_, 4 mM ribonucleotide triphosphates (rNTPs), 1× transcription buffer (Thermo Fisher), 2.5 to 5 μg DNA, and 0.05 mg/ml T7 RNA polymerase (Thermo Fisher) and in a 50-μl reaction volume, with incubation at 37°C for 1 h. The RNA was treated with proteinase K (Sigma) and DNase I (Sigma) according to manufacturer’s instructions. Afterward, the RNA was isolated by successive rounds of EtOH precipitation, and the dried pellet was resuspended in double-distilled water (ddH_2_O) at the desired concentrations.

### EMSA.

Electrophoretic mobility shift assay (EMSA) was performed as described previously (37). DNA was from ThermoFisher or produced by PCR amplification with pCF10 Mini or pRS01 as the template (IGR and control DNA). DNA (100 nM) was mixed with increasing concentrations of PrgU with an N- (Fig. S2) or C-terminal (Fig. 7) His tag. Binding was allowed to occur for 20 min at room temperature (∼25°C), and the complexes were then run at 4°C on 1% agarose gels at 100 V for 40 min. The visualization was done by poststaining with 3× GelRed and recording on a ChemiDoc (Bio-Rad).

### MST.

The microscale thermophoresis (MST) was performed as follows. PrgU-His was labeled with the Red second-generation His tag labeling kit (NanoTemper Technologies) according to the manufacturer’s instructions with a final concentration in the assay of 50 nM (molar dye/protein ratio of 4:1). A 16:1 dilution series was performed with RNA of IGR and a control sequence of the same length, with 40 μM as the highest concentration in the assay. The samples were mixed in a buffer containing 20 mM KPi (pH 7.0), 150 mM NaCl, 0.05% Tween 20, and 0.1 U/μl Protector RNase inhibitor (Sigma-Aldrich) and incubated for 10 to 20 min at room temperature. After incubation, the samples were quickly spun down and loaded into Monolith NT.115 standard capillaries (NanoTemper Technologies) at room temperature (∼25°C). MST measurements were carried out with a Monolith (Monolith NT.115) using the instrument parameters of 60 to 100% LED power and medium MST power, the Red filter, and an MST on time of 20 s. Data were collected and analyzed using MO.Monolith Control software V1.6.1 and MO.Monolith Affinity Analysis V2.3. Three biological replicates were compared for each sample. Figures were made in GraphPad Prism 8.0.

### Data availability.

The data that support the findings of this study are available in the supplemental material of this article. RNA-seq data have been deposited with NCBI GEO under accession numbers GSE163794 and GSE168958.
