# Supplementary figures and images for: Enterococcal PrgU Provides Additional Regulation of Pheromone-Inducible Conjugative Plasmids
Source: mSphere. 2021 Jun 9;6(3):e00264-21. doi: 10.1128/mSphere.00264-21 (PMC8265641; doi:10.1128/mSphere.00264-21)

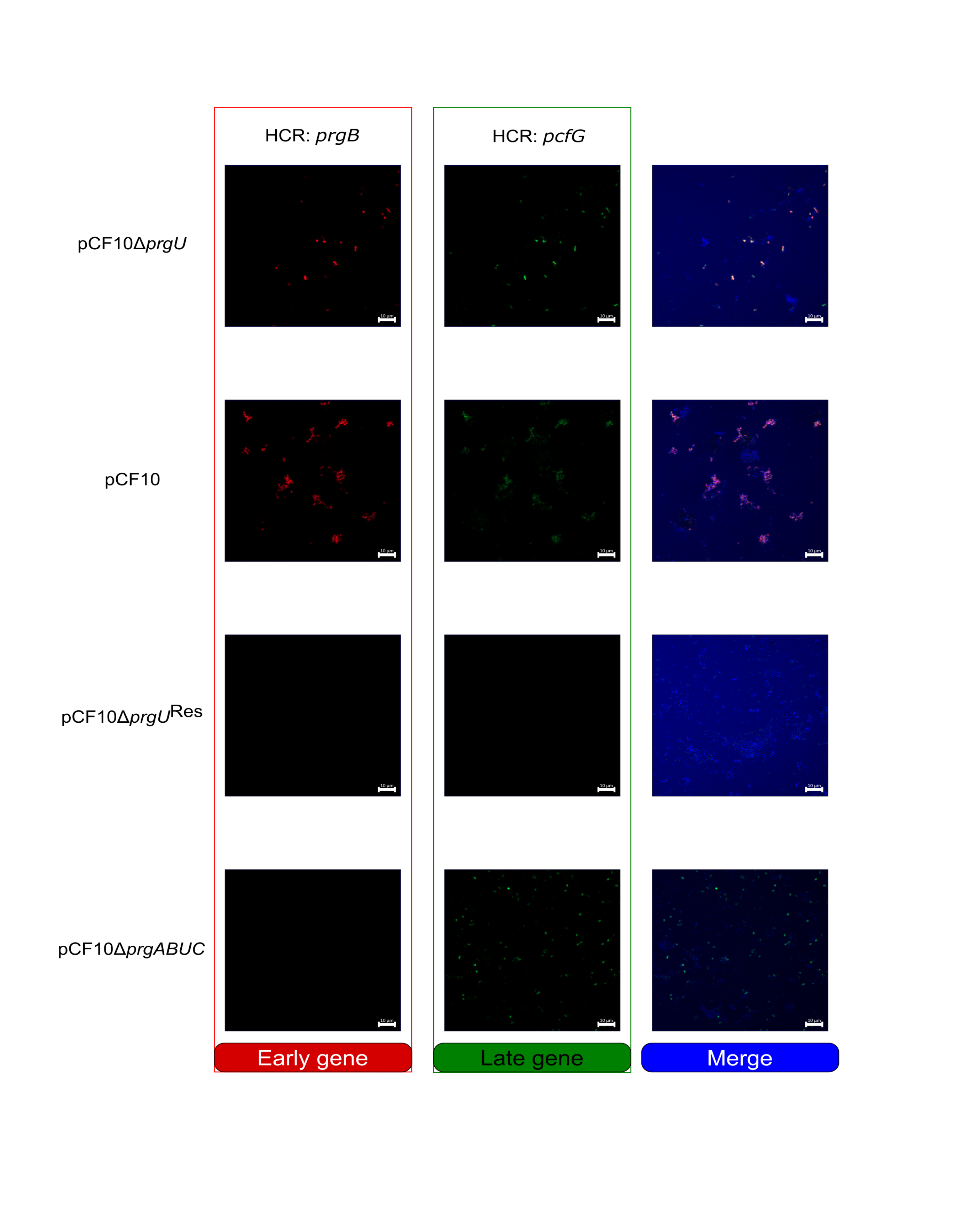

Supplement: FIG S1 [file msphere.00264-21-sf001.tif]

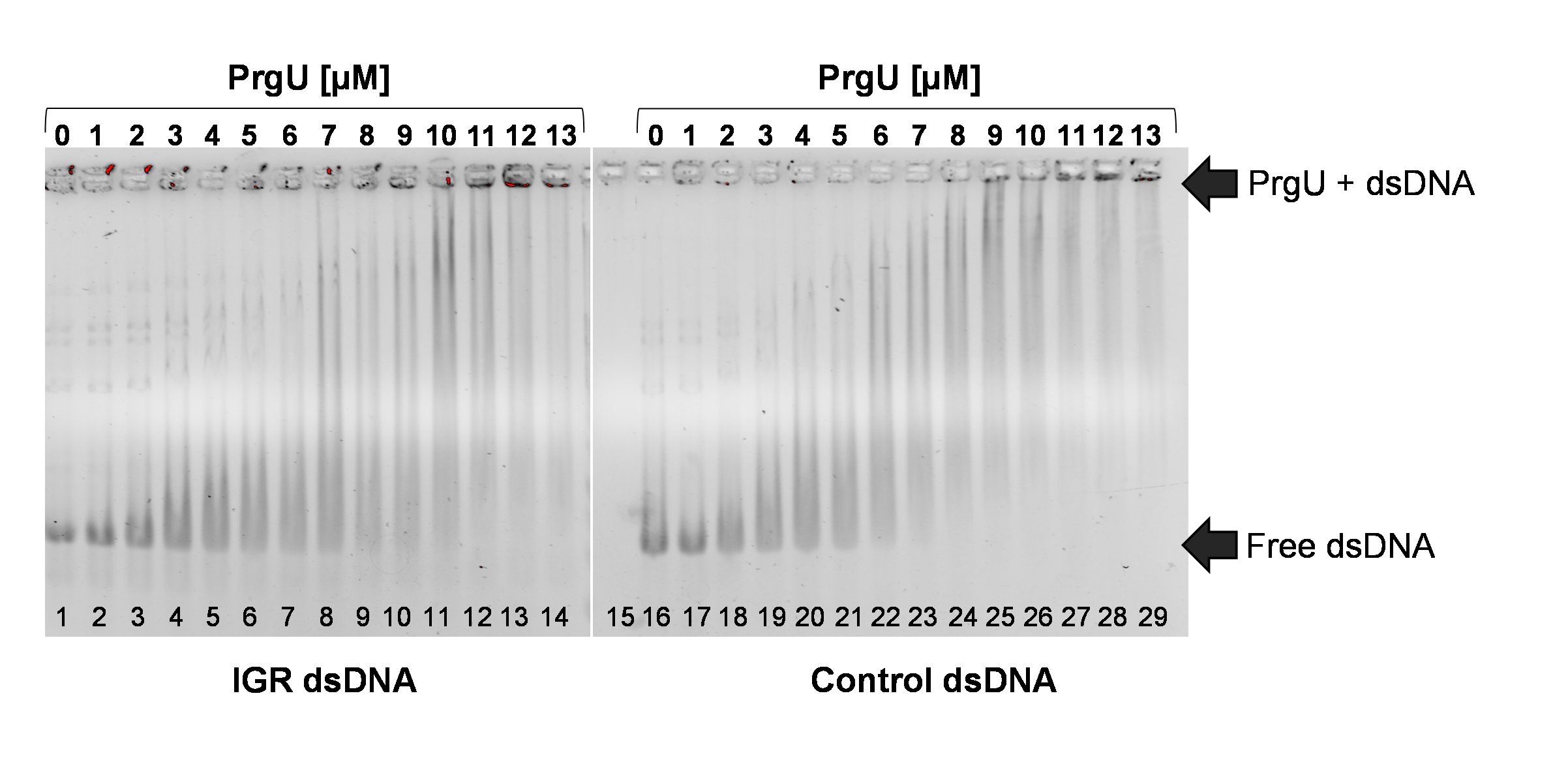

Supplement: FIG S2 [file msphere.00264-21-sf002.tif]
