# Supplementary material for: Enterococcal PrgU Provides Additional Regulation of Pheromone-Inducible Conjugative Plasmids
Source: mSphere. 2021 Jun 9;6(3):e00264-21. doi: 10.1128/mSphere.00264-21 (PMC8265641; doi:10.1128/mSphere.00264-21)
Supplement: TABLE S1 [file msphere.00264-21-st001.docx]

| Oligo | Sequence |
| --- | --- |
| IRS1 ssDNA | GGAGCAGCGGGGAATGTATACAGTTCATGTATATGTTCCCCGCTTTTTTTG |
| IRS1 complementary strand ssDNA | CCTCGTCGCCCCTTACATATGTCAAGTACATATACAAGGGGCGAAAAAAAC |
| IRS1 RNA | GGAGCAGCGGGGAAUGUAUACAGUUCAUGUAUAUGUUCCCCGCUUUUUUUG |
| IRS2 ssDNA | ACTCGTAAGAGCCGTGCAAACAACGTGTTGCATGGCTCTTTTTCTAC |
| IRS2 complementary strand ssDNA | TGAGCATTCTCGGCACGTTTGTTGCACAACGTACCGAGAAAAAGATG |
| IRS2 RNA | ACUCGUAAGAGCCGUGCAAACAACGUGUUGCAUGGCUCUUUUUCUAC |
| Control ssDNA | TCAGGGAGTTTAAGTCGAGTCAATAGAGCTCGCAATACAGAGTTTACCGC |
| Complementary strand ssDNA | AGTCCCTCAAATTCAGCTCAGTTATCTCGAGCGTTATGTCTCAAATGGCG |
| Control RNA | UCAGGGAGUUUAAGUCGAGUCAAUAGAGCUCGCAAUACAGAGUUUACCGC |
