# Supplementary material for: Enterococcal PrgU Provides Additional Regulation of Pheromone-Inducible Conjugative Plasmids
Source: mSphere. 2021 Jun 9;6(3):e00264-21. doi: 10.1128/mSphere.00264-21 (PMC8265641; doi:10.1128/mSphere.00264-21)
Supplement: TABLE S2 [file msphere.00264-21-st002.docx]

| **Strains or plasmids** | **Relevant features** | **Source or Reference** |
| --- | --- | --- |
| **Strains** |  |  |
| *E. coli* |  |  |
| DH5α | F-φ80d*lacZ* ΔM15 Δ(*lacZYA-argF*)U169 *deoR recA1 endA1hsdR17(rK-mK+)phoA supE44λ- thi-1gyrA96 relA1* | Gibco-BRL |
|  |  |  |
| *E. faecalis* |  |  |
| OG1RF | Rif^r^ Fus^r^ | (1) |
| OG1ES | Ery^r^ Str^r^ | (2) |
| **Plasmids** |  |  |
| pCJK47 | Spc^r^, carries *oriT*_pCF10,_ *lacZ,* and P-pheS* cassette | (3) |
| pDL278p23 | Spc^r^, pDL278 with *L. lactis* constitutive promoter P_23_ | (4) |
| pCI372 | Chl^r^, *E. coli* - *E. faecalis* shuttle vector | (5) |
| pCF10 | Tet^r^, pheromone inducible conjugative plasmid | (6) |
| pCJK205 | Erm^r^, plasmid constitutively expressing *lacZ* | (7) |
| pCIE | Chl^r^, pCI372 with the pCF10 regulatory region | Dunny Lab |
| pCF10Δ*prgU* | Tet^r^, pCF10 deleted of *prgU* | (8) |
| pCF10Δ*prgU*^RES^ | Tet^r^, pCF10 deleted of *prgU* and *prgR* Δ271 | (8) |
| pCF10Δ*prgABUC* | Tet^r^, pCF10 deleted of *prgA prgB, prgU* and *prgC* | (9) |
| p10-mini | Chl^r^, pCI372 carrying the entire *prgQ* regulatory region and the *prgA-prgC* gene cassette | (8) |
| pMB11 | Spc^r^, pDL278p23 expressing P_23_::*prgU* | (8) |
| pMC1 | Spc^r^, pCI372 carrying *prgX* - p*rgA* from pCF10 followed by *lacZ* with its Shine-Dalgarno sequence. | This study |
| pMC2 | Spc^r^, pMC1 carrying *prgX - 5’ prgR* from pCF10 with *lacZ* at the *prgR* start-site. | This study |
| pMC3 | Spc^r^, pMC2 deleted of the IGR sequence,  *lacZ* is located immediately after *prgQ* | This study |
| pMC9 | Chl^r^, pMC2 deleted of *prgX* | This study |
| pMC10 | Spc^r^, pDL278p23 expressing P_23_::*prgU-FLAG* | This study |
| pMC11 | Spc^r^, pDL278p23 expressing P_23_::*IGR* | This study |
| **Primers** | **Sequence (5’ to 3’)** | **Used for** |
| PrgU_flagCT_F | GACGATGACAAATAAGCATGCAAGCTTGGCGTAATC | pMC10 |
| PrgU_flagCT_R | ATCTTTATAATCTGATTTTAAAGTTTCGCCGAAGATCTC | pMC10 |
| pCIE_LacZ_F | AATCGAGGAGAATGATACATGAATCGG | pMC2, pMC3 |
| pCIE_XQ_inter_R | CATTGATTCACCTACTTTCTGAACCAAG | pMC2 |
| pCIE_XQ_R | CTATCAGATAAATATTAAGGTTATTGCAATTACAAC | pMC3 |
| pCIE_prgX_F | CATTTTCTTCCTCCTAATATCTCGAGTATC | pMC9 |
| pCIE_prgX_R | CCCCCTAAAGAAGTAACCATGTATTATG | pMC9 |
| EcoRI_T7_IGR_F | GCTATCGAATTCTAATACGACTCACTATAGGGCTAGAAAAAATCATAGTAACAATTAAAC | Cloning into pRAV23 |
| HindIII_IGR_R | GGGCCAAAGCTTTGATTCACCTACTTTCTGAAC | Cloning into pRAV23 |
| EcoRI_T7_LtrD_F | AATATCGAATTCTAATACGACTCACTATAGGGCATGTCAATGAATGAAGCAGATTTT | Cloning into pRAV23 |
| HindIII_LtrD_R | GGGCCAAAGCTTCTACTTGCGATTTTTTCTTTTTCC | Cloning into pRAV23 |
| **Anti-Sense Oligonucleotides for Northern Blots** |  |  |
| Anti-IGRa | [Btn]GTTGCAACAAACGAGAACCGAGTAGAGTTCATGTC |  |
| Anti-5S | [Btn}GGGAACAGGTGTATCCTTCTCGCTATCGCCACCAC |  |

**Supplementary References**

1. Dunny GM, Brown BL, Clewell DB. 1978. Induced cell aggregation and mating in Streptococcus faecalis: evidence for a bacterial sex pheromone. Proc Natl Acad Sci U S A 75:3479–3483.

2. Staddon JH, Bryan EM, Manias DA, Chen Y, Dunny GM. 2006. Genetic characterization of the conjugative DNA processing system of enterococcal plasmid pCF10. Plasmid 56:102–111.

3. Kristich CJ, Chandler JR, Dunny GM. 2007. Development of a host-genotype-independent counterselectable marker and a high-frequency conjugative delivery system and their use in genetic analysis of Enterococcus faecalis. Plasmid 57:131–144.

4. Chen Y, Staddon JH, Dunny GM. 2007. Specificity determinants of conjugative DNA processing in the Enterococcus faecalis plasmid pCF10 and the Lactococcus lactis plasmid pRS01. Mol Microbiol 63:1549–1564.

5. Hayes F, Daly C, Fitzgerald GF. 1990. Identification of the minimal replicon of Lactococcus lactis subsp. lactis UC317 plasmid pCI305. Appl Environ Microbiol 56:202–209.

6. Dunny G, Funk C, Adsit J. 1981. Direct stimulation of the transfer of antibiotic resistance by sex pheromones in Streptococcus faecalis. Plasmid 6:270–278.

7. Djorić D, Kristich CJ. 2015. Oxidative stress enhances cephalosporin resistance of Enterococcus faecalis through activation of a two-component signaling system. Antimicrob Agents Chemother 59:159–169.

8. Bhatty M, Camacho MI, González-Rivera C, Frank KL, Dale JL, Manias DA, Dunny GM, Christie PJ. 2016. PrgU: A Suppressor of Sex Pheromone Toxicity in Enterococcus faecalis. Mol Microbiol 103:398–412.

9. Bhatty M, Cruz MR, Frank KL, Gomez JAL, Andrade F, Garsin DA, Dunny GM, Kaplan HB, Christie PJ. 2015. Enterococcus faecalis pCF10-encoded surface proteins PrgA, PrgB (aggregation substance) and PrgC contribute to plasmid transfer, biofilm formation and virulence. Mol Microbiol 95:660–677.
